# The Role of miRNAs in Virus-Mediated Oncogenesis

**DOI:** 10.3390/ijms19041217

**Published:** 2018-04-17

**Authors:** Zuzana Vojtechova, Ruth Tachezy

**Affiliations:** Department of Genetics and Microbiology, Faculty of Science, Charles University, Průmyslová 595, Vestec, CZ-25250, 128 44 Prague, Czech Republic; zuzana.vojtechova@natur.cuni.cz

**Keywords:** microRNA, virus-mediated oncogenesis, viral miRNA, EBV, HHV-8, HBV, HPV, MCPyV, HCV, retroviruses

## Abstract

To date, viruses are reported to be responsible for more than 15% of all tumors worldwide. The oncogenesis could be influenced directly by the activity of viral oncoproteins or by the chronic infection or inflammation. The group of human oncoviruses includes Epstein–Barr virus (EBV), human papillomavirus (HPV), hepatitis B virus (HBV), hepatitis C virus (HCV), human herpesvirus 8 (HHV-8) or polyomaviruses, and transregulating retroviruses such as HIV or HTLV-1. Most of these viruses express short noncoding RNAs called miRNAs to regulate their own gene expression or to influence host gene expression and thus contribute to the carcinogenic processes. In this review, we will focus on oncogenic viruses and summarize the role of both types of miRNAs, viral as well as host’s, in the oncogenesis.

## 1. Introduction

More than 14 million cancer cases are diagnosed each year worldwide, with more than 15% attributable to the carcinogenic infections [1]. Oncoviruses are responsible for more than 63% of cases, the bacterium *Helicobacter pylori* accounts for 35%, and the remaining 2% of cases are associated with three parasites (*Schistosoma haematobium*, *Opisthorchis viverrini* and *Clonorchis sinensis*). In 2000, Hanahan and Weinberg identified six hallmarks of cancer—evading antigrowth signals, tissue invasion and metastasis, enabling replicative immortality, sustained angiogenesis, evading apoptosis, and maintaining proliferative signaling [2]. In 2011, these principles were updated by adding another four, including deregulated cellular energetics, avoiding of immunological destruction, tumor-promoting inflammation, and genomic instability and mutations [3]. Oncoviruses, as infectious agents, can induce all of these hallmarks and thus contribute to tumor development through multiple pathways. However, it is important to mention that in viral oncogenesis, viruses are necessary but not sufficient to cause cancer, so the incidence of cancer is much lower than the prevalence of the causative viruses [4].

The oncogenic viruses evoke and maintain persistent infection during which they are hidden from the immune system, which is compatible with carcinogenic processes. Most oncogenic viruses have mechanisms through which they are equally segregated into the daughter cells during cell division, and thus their genome is maintained in the host cells during proliferation. The virus-mediated cell immortalization is then influenced either directly or indirectly. The direct mechanisms include the deregulated expression of cellular oncogenes/tumor-suppressor genes, influenced by integration of the viral genome into the host genome (e.g., retroviruses, human papillomaviruses, hepatitis B viruses), or the expression of viral oncogenes (e.g., herpesviruses) which inactivate major regulators of genome stability and cell cycle leading to DNA damage and transformation of the host cell. The indirect mechanisms of transformation comprise the tissue damage caused by immune cells and chronic inflammation, or establishment of immunosuppression due to viral infection, resulting in the inhibition of antitumor surveillance mechanisms.

Oncogenic viruses are represented in all groups of viruses. The most relevant are DNA viruses, of which we should mention Epstein–Barr Virus (EBV), human papillomavirus (HPV), hepatitis B virus (HBV), human herpesvirus 8 (HHV-8), or polyomaviruses. The group of RNA viruses is represented by hepatitis C virus (HCV), and finally, the group of retroviruses includes transregulating viruses such as human immunodeficiency virus (HIV) or human T-lymphotropic virus (HTLV-1).

MicroRNAs (miRNAs) are short noncoding RNAs of ~21 nucleotides in length which post-transcriptionally regulate gene expression and have an important role in the development, cell growth, differentiation processes, survival, or regulation of apoptosis in a variety of eukaryotic organisms [5]. It is suggested that the expression of at least one third of human genes is influenced by miRNAs [6], and the deregulated expression of miRNAs has been observed in many types of tumors [7,8]. However, most of the oncogenic viruses also express miRNAs to regulate their own gene expression or to influence host gene expression, and thus contribute to the carcinogenic processes. Even though many new virus-encoded miRNAs were discovered in the last 15 years, only a minority of them were shown to fulfill the criteria for authentic viral miRNAs. These criteria are very important when deep-sequencing techniques are applied, since many small non-viral RNAs can be detected. Besides the number of miRNA copies per cell, identification of the region in the viral genome from which the miRNA is derived, the size of the miRNA within the range of 19–25 bases, the specificity of the 5′ end, and identification of the hairpin structure of pre-miRNA should be specified for an authentic viral miRNA [9]. In this review, we will more closely focus on the role of viral miRNAs in oncogenesis and on the impact of their expression on the hosts. Moreover, the role of the host’s miRNA expression during oncogenesis will be discussed.

## 2. Epstein–Barr Virus

Epstein–Barr Virus (EBV, HHV-4), the first oncovirus discovered, is classified as a DNA virus from the family *Herpesviridae*. It was detected first in Burkitt lymphoma (BL) cells in 1964 [10], and this discovery started the research on the virus-mediated oncogenesis. The primary infection of EBV is most common in childhood, and then EBV persists in latent form mostly in resting memory B-cells and less commonly in T-cells, NK-cells, or epithelial cells [11]. Besides Burkitt lymphoma, EBV is associated with other B-cell lymphoproliferative disorders such as Hodgkin lymphoma (HL) or post-transplant lymphoproliferative disorder (PTLD), with T-cell lymphoproliferative disorders or epithelial malignancies such as gastric carcinoma and nasopharyngeal carcinoma (NPC) [12,13,14].

The mechanism of EBV-mediated carcinogenesis is the coding of the viral oncoproteins. The main oncoprotein is latent membrane protein 1 (LMP1), which is a transmembrane protein functionally mimicking CD40, a member of the tumor necrosis factor receptor (TNFR) superfamily. The activation of this receptor leads to the initiation of the signaling pathways such as phosphoinositide 3-kinase/protein kinase B (PI3K/Akt), mitogen-activated protein kinase (MAPK), Janus kinase/signal transducer and activator of transcription proteins (JAK/STAT) or nuclear factor kappa-light-chain-enhancer of activated B cells (NF-κB), leading to B-cell differentiation towards memory B-cells, expression of anti-apoptotic proteins, and increased cell proliferation [15]. Oncoprotein LMP2 is structurally and functionally similar to the B-cell receptors (BCRs) which, after phosphorylation, activate the non-receptor tyrosine kinase (Src) and spleen tyrosine kinase (Syk) signaling pathways and thus increases the survival of latently infected B-cells by production of cytokines such as interleukin 10 (IL-10) and by expression of anti-apoptotic factors [16,17,18]. EBV nuclear antigen 1 (EBNA1) is a multifunctional protein which influences viral replication, transcription and latency. As the only one of the nuclear proteins, it is expressed in both lytic and latent phases of the viral life cycle [19]. EBNA1 suppresses the function of the promyelocytic leukemia (PML) protein, which is a tumor suppressor protein regulating p53 activation. Thus, EBNA1 inhibits p21 activation and signaling, leading to inhibition of apoptosis and cell survival [20]. EBNA-LP (EBNA leader protein) functions in cooperation with EBNA2, and both proteins participate in initiating the transcription of viral and cellular proteins responsible for B-cell immortalization and transformation, for example, cellular gene *c-myc* [21]. Finally, EBNA-3 proteins associate with many cellular proteins from different signaling pathways such as recombination signal binding protein for immunoglobulin kappa J region (RBP-Jκ) and thus contribute to increased proliferation and transformation of B-cells [22].

EBV was the first virus for which the viral-encoded miRNAs were described. Pfeffer et al. have published the report describing viral-encoded miRNAs from cell lines infected with EBV [23]. These five miRNAs were encoded in two clusters, BHRF1 (miR-BHRF1-1, miR-BHRF1-2, miR-BHRF1-3) and BART (miR-BART1 and miR-BART2). Nowadays, according to the miRBase database, EBV encodes 25 pre-miRNAs producing at least 44 mature miRNAs [24]. The benefit of viral-encoded miRNAs, not only for EBV but for all viruses coding their own miRNAs, is the ability to regulate viral as well as host gene expression without the production of viral proteins, allowing for the immune invisibility of the infected cells.

As mentioned above, EBV miRNAs have been reported to regulate their own expression. BART miRNAs (miR-BART16, -17-5p, -1-5p) target viral transcripts for protein LMP1 and thus contribute to cell transformation [25]. MiR-BART22 negatively regulates translation of LMP2A in NPC and thus helps EBV-infected cells to escape the host immune surveillance [26]. EBV also uses the miRNAs for indirect regulation of the switch between the lytic and latent life phases. For example, miR-BART2 regulates DNA polymerase BALF5 and helps to replicate the viral genome in the lytic phase, and thus its negative regulation promotes the latent stage of the virus [27]. Iizasa et al. have reported miR-BART6-5p to regulate the viral replication and latency through suppressing the EBNA2 viral oncogene [28].

EBV miRNAs also regulate the translation of host mRNAs and thus may influence cancer development. MiR-BART5-5p and miR-BART19-5p negatively regulate the translation of p53 upregulated mediator of apoptosis (PUMA) protein, a factor that positively influences cellular apoptosis and was found at significantly lower levels in NPC cells infected by EBV compared to noninfected ones [29]. Nasopharyngeal carcinoma cells were analyzed also by Cai et al. [30], and they revealed that miR-BART7-3p promotes the epithelial–mesenchymal transition (EMT) and metastasis through suppressing the major human tumor suppressor PTEN, which modulates PI3K/Akt/GSK-3β signaling. Vereide et al. revealed that miR-BART1 and miR-BART16 help to block apoptosis in BL cells in the absence of other viral oncogenes through their targeting of caspase 3 (Casp3) [31]. Infected cells thus survive, and the virus may replicate. Further, the EBV miRNA miR-BART3 has been reported to negatively regulate the expression of the cellular tumor suppressor DICE1 (determination of interleukin 4 commitment 1), which was found downregulated in NPC cells, leading to increased proliferation and transformation of the cells [32]. EBV also uses its own miRNAs for the immune evasion strategy. MiR-BHRF1-3 targets the mRNA of host interferon-inducible cytokine CXCL11 [33]. Targeted suppression of this cytokine may serve as an immunomodulatory mechanism in EBV-associated tumors. In-vitro studies have demonstrated that the action of miR-BHRF1 enhances the B-cell transformation and decreases the antigen loading of cells [34]; however, in-vivo studies have revealed that miR-BHRF1 facilitates the development of acute infection but does not enhance the oncogenic potential [35]. The evasion of the host immune system is also mediated by miR-BART2-5p, which negatively regulates the expression of major histocompatibility complex (MHC) class I chain-related protein B (MICB) molecules upregulated on the infected cells’ surface and recognized by NK cells [36]. The expression of EBV-encoded miRNAs may also influence the survival of patients with EBV-associated cancer. A high level of miR-BART20-5p was associated with worse survival of patients with EBV-related gastric cancer [37]. Moreover, the expression of viral miR-BART7 predicted the responsiveness of NPC cells to radiation treatment because of its targeting of the glutamine fructose-6-phosphate transaminase 1/transforming growth factor beta 1 (GFPT1/TGFβ1) signaling, regulating the DNA damage repair machinery [38].

The deregulation of cellular miRNA expression is a feature detectable in many human tumors including EBV-associated tumors. It has been reported that EBV infection of primary B-cells results in downregulation of cellular miRNA expression [39]. Iizasa et al. have revealed that EBV miR-BART6-5p suppresses the expression of Dicer and thus influences expression of many miRNAs as well as miR-BART6-5p itself by a negative feedback loop [28]. The tumor suppressor miR-31 is consistently inactivated in NPC, since it targets the MCM2 protein and inhibits the growth of NPC cells [40]. Nevertheless, many cellular miRNAs are upregulated due to the infection, such as miR-155, which promotes the proliferation and migration of NPC cells [41] or B-cell immortalization [42]. The aberrant expression of miR-155 is driven by EBV LMP1 and LMP2A [43,44], and its upregulated expression in EBV-associated tumors was also confirmed by the study of Sakamoto et al. [45], who used a next-generation sequencing approach. The miRNA related to EBV infection in BL is miR-127, which was not found in EBV-negative BL, and it contributes to the development of lymphoma through the blocking of BLIMP-1 or XBP-1, regulators of B-cell differentiation [46]. MiR-21, a very important oncomiR in tumor development, was found to be positively regulated by EBV EBNA2 protein in B-cell lymphoma [47], and in tumors has anti-apoptotic and prometastatic roles. The EBNA2 protein further negatively regulates the expression of miR-146a, a miRNA involved in the innate immune response [47]. Oussaief et al. [48] have shown that LMP1 protein expression triggered downregulation of the miR-183-96-182 cluster, whose expression is downregulated in BL cell lines and has a key role in EBV-mediated transformation. Finally, Chen et al. [49] have revealed that the expression of miR-1 in NPC is downregulated by LMP1. This miRNA functions as a tumor suppressor by regulating *K-ras* (Kirsten rat sarcoma) gene expression with pro-apoptotic effect and inhibition of the angiogenesis during tumor development.

## 3. HHV-8

Another oncovirus from the family *Herpesviridae* is human herpesvirus 8 (HHV-8). Sometimes it is called Kaposi’s sarcoma-associated herpesvirus (KSHV) since it is a causal agent of Kaposi’s sarcoma (KS), a proliferative disease of vascular and lymphatic endothelial cells [50]. HHV-8 infection mainly manifests in immunocompromised patients, such as those with AIDS and those after transplantation or chemotherapy. Apart from KS, HHV-8 causes primary effusion lymphoma (PEL) [51] or multicentric Castleman’s disease [52], with both affecting B-cells. HHV-8 influences the proliferation and cell cycle of the infected cells due to the sequence homology with host genes. In the latent phase of the viral infection, viral cyclin D (v-cyclin D) and LANA1 regulating the cell cycle, viral FLICE inhibition protein (v-FLIP)—inhibitor of apoptosis, kaposins, or viral interferon response factors (vIRF) modulating the immune system and influencing the proliferation—are expressed.

KSHV encodes 12 viral pre-miRNAs, which evolve into 25 mature miRNAs. All miRNA genes are clustered together and are under the control of latent kaposin promoter (LTd). Most of the pre-miRNA genes are intronic, located between the sequence for *kaposin* and open reading frame (ORF) 71, except for miR-K10, which is located within the ORF of *kaposin*, and miR-K12 located on the 3′ end of the *kaposin* gene [53]. It is important to mention that viral latency is critical for tumor development. KSHV miRNAs participate in the regulation of the viral life cycle targeting directly key viral genes as well as indirectly, through cellular genes regulating viral replication and thus contributing to the oncogenesis. The viral miRNAs miR-K9-5p and miR-K7-5p are modulators of the latent–lytic switch as they target the viral RTA (R transactivator) protein, the regulator of lytic induction [54,55]. The viral life cycle is also regulated by miR-K3, which targets cellular nuclear factor I/B and thus negatively influences the expression of RTA [56], or by G-protein-coupled receptor kinase 2 (GRK2), enhancing in this way the viral latency [57]. The expression of RTA could also be restrained by the activity of miR-K12-11 which targets cellular myeloblastosis transcription factor (MYB) [58], previously reported to be an activator of the RTA promoter [59]. Moreover, miR-K12-11 modulates interferon signaling through targeting I-kappa-B kinase epsilon (IKKε), contributing to the maintenance of viral latency [60]. The latent phase of the viral infection is also maintained by methylation of the *RTA* promoter, which is ensured by DNA methyl transferase 1 (DNMT1) [61]. The activity of this methyltransferase is regulated by KSHV miR-K12-4-5p, which inactivates its suppressor retinoblastoma-like protein 2 (Rbl2), affecting cell cycle and cellular differentiation control.

KSHV miRNAs play roles also in the dissemination and angiogenesis of KS. Several miRNAs, such as miR-K12-1, miR-K3-3p, miR-K6-3p, or miR-K12-11, negatively regulate the expression of thrombospondin 1 (THBS1), which is an antagonist of angiogenesis, and its downregulation leads to abnormal angiogenesis and proliferation of KSHV-infected cells [62]. Another miRNA promoting dissemination and angiogenesis is miR-K6-3p, whose activity stimulates the STAT3 pathway leading to cell migration and the invasion of KS cells [63]. As has been shown by Guo et al. [64], KSHV miRNAs regulate matrix metalloproteinases (MMPs) and expression of pro-angiogenic factors, and thus play roles in KSHV-induced cell motility and angiogenesis. KSHV miRNAs help to promote the development of KS and other KSHV-associated malignancies through the cell cycle arrest, cell survival, and cell transformation. Zhu et al. have reported that KSHV promotes these processes by suppressing the aerobic glycolysis and oxidative phosphorylation under nutrient stress [65]. They have revealed that KSHV regulates the key metabolic pathways of the cells by miRNAs to adapt to the tumor microenvironment. MiR-K12-1 is an anti-apoptotic miRNA, which downregulates the expression of protein p21, the inhibitor of cyclin-dependent kinases and a key inducer of cell cycle arrest [66], and in this way, contributes to the survival of viral-transformed cells. Also, miR-K12-1, miR-K12-3, and miR-K12-4-3p, which inactivate the critical inducer of apoptosis, caspase 3 (Casp3), participate in inhibition of apoptosis and thus play a role in KSHV-induced oncogenesis [67].

Like EBV, KSHV influences the expression and function of cellular miRNAs. Viral miR-K12-11, playing a role in PEL development, shares the seed sequence with the cellular miR-155, whose targets affect B-cell differentiation, and thus regulates a set of common mRNA targets [68,69]. Tsai et al. have revealed that KSHV protein K15 via the viral SH2-binding motif contributes to KSHV-associated tumor metastasis and angiogenesis by regulation of cellular miR-21 and miR-31 [70]. The KSHV-encoded protein vFLIP K13 activates the NF-κB pathway, suppressing the expression of cytokine C-X-C chemokine receptor type 4 (CXCR4) through upregulation of cellular miR-146a [71]. CXCR4 plays a key role in the retention of immature endothelial cells in the marrow, and its downregulation contributes to premature release of these cells into the circulation and to KS development. Only one study analyzing the miRNA expression profiles in KSHV-infected B-cells has been published so far. Hussein and Akula performed the analysis of the early stages of KSHV infection of human B-cells and have revealed 32 known and 28 novel differentially expressed miRNAs [72]. The potential biological implications of the known differentially expressed miRNAs included promoting cell survival and latent infection, inhibiting the host immune response, or inducing critical cell signaling.

## 4. Hepatitis B Virus

Hepatitis B virus (HBV) belongs to the family *Hepadnaviridae* and causes an acute liver disease—viral hepatitis. Most infected adults recover completely in a few months, but about 5% of the adult patients proceed to chronic infection [11], which can consequently develop into hepatocellular carcinoma (HCC). In patients infected perinatally or in childhood, the percentage of chronic infection is higher (90% and 20% of cases, respectively). HBV is the third most frequent infectious agent contributing to cancer development, with 420,000 of new cancer cases reported in 2012 [1]. Apart from HCC, HBV infection is associated with B-cell non-Hodgkin lymphoma (B-NHL) [73] and nasopharyngeal carcinoma (NPC) [74], but the exact virus-associated pathogenesis is still unclear.

The HBV genome encodes four ORFs, which consist of genes for surface proteins (*preS1*, *preS2* and *S*), genes for core (*core* and *precore*) proteins, genes for polymerase, and genes for protein HBx. The oncogenesis is influenced by the direct mechanisms of the virus such as activity of viral oncoprotein HBx or surface proteins in the transcriptional regulation, regulation of DNA repair or expression of miRNAs [75,76,77,78], and/or the integration of the viral DNA into the host genome in the proximity of the fragile sites [79,80]. Further, HBV contributes to the development of the tumors by indirect mechanisms, such as chronic inflammation and activation of protumorigenic signaling pathways [81].

The presence and the function of HBV-encoded miRNAs has so far not been proven, but at least two published studies suggested existence of HBV-specific miRNAs, and we discuss them below. On the other hand, numerous studies focused on the cellular miRNA profiles and their deregulation during HBV-associated tumorigenesis have been described. Highly specific to hepatocytes is miR-122, which maintains the differentiated phenotype of cells, and its downregulation was observed in HCC cell lines as well as in clinical samples. MiR-122 negatively regulates the expression of a tumor promoter, N-myc downstream-regulated gene 3 (*NDRG3*) [82], or pituitary tumor-transforming gene 1 (PTTG1) binding factor (PBF) whose upregulation, because of the loss of miR-122 expression, leads to the cell growth and invasion of the HCC tumor [83]. The tumor cell growth is also regulated by cyclin G1, whose expression increases depending on the suppression of miR-122 [84]. Song et al. [85] have revealed that the reduction of the miR-122 level in HCC cells is due to the protein–protein activity of viral HBx protein with peroxisome proliferator activated receptor-gamma (PPARγ), which normally enhances the miR-122 transcription by binding to its promoter. Besides miR-122, protein HBx further influences the expression of cellular miR-29a, which is upregulated in HBx-transfected hepatoma cells and whose upregulation positively correlates with the metastatic potential [86]. MiR-29a increases the migration ability of cells through targeting tumor suppressor phosphatase and tensin homolog (PTEN) and activation of the Akt signaling pathway. MiR-29a/b also targets MHC class I chain-related protein A (MICA) or MICB, whose decreased expression results in a limited activity of natural killer (NK) cells and promotion of chronic infection [87]. Moreover, HBx decreases the level of miR-101, enhancing tumorigenesis through epigenetic silencing of tumor suppressor genes (*TSGs*) [88]. This miRNA targets DNA methyltransferase 3A (DNMT3A), which catalyzes the methylation of *TSG* promoter regions and thus inhibits their expression. DNA methylation is also influenced through HBx downregulation of miR-152, which targets DNA methyltransferase 1 (DNMT1) and epigenetically regulates the expression of *TSGs* [89]. Not only miRNAs mediate epigenetic modifications of DNA, but also the protein HBx itself induces DNA methylation of promoter. Wei et al. [90] have revealed that HBx induces DNA hypermethylation of the miR-132 promoter and thus promotes cell proliferation through the Akt signaling pathway. Moreover, they have found that the serum levels of miR-132 correlate with that in the tumor tissues, implicating that it might be a noninvasive candidate for a diagnostic biomarker of HBV-related HCC. Protein HBx also negatively regulates the level of miRNA let-7a [91]. These tumor suppressor miRNAs are involved in cell differentiation and proliferation through the STAT signaling pathway, and are often downregulated in HCC. Also, a decreased level of let-7a leads to the activation of proliferation factors, such as Ras [92] or Myc [93]. Let-7 expression might also be regulated by HBx indirectly through the upregulation of the let-7 inhibitors LIN28A and LIN28B [94,95].

Profiling studies of virus-related HCC search for miRNAs specific for the early stages of the tumors or progression of the disease. Mizuguchi et al. [96] have used next-generation sequencing and bioinformatics to reveal the miRNA transcriptome of HBV-related HCC. The global profiling of miRNAs in HBV-related HCC was also performed by Wang et al. [97], who analyzed 12 pairs of HCC and matched nonmalignant tissues from HBV-positive and HBV-negative patients. They have revealed eight miRNAs involved in HCC unrelated to virus, a further five miRNAs involved in HBV infection, and finally, seven miRNAs specifically altered in HBV-associated HCC. The possible role of these miRNAs (miR-150, miR-342-3p, miR-663, miR-20b, miR-92a-3p, miR-376c-3p, and miR-92b) in HBV-related HCC development must be further investigated.

Not only viruses can influence the expression of cellular miRNAs and thus the development of the tumor, but also host miRNAs regulate the viral life cycle and replication and thus affect the progression of chronic hepatitis to HCC. Direct targeting of HBV mRNA, by transcript of gene *HBx*, was observed by Wang et al. [98]. They have found that the tumor suppressors miR-15a/miR-16-1 target viral mRNA and thus reprogram the expression of multiple cellular miRNAs including these miRNAs themselves, leading to the HCC development. A similar negative feedback suppression was observed by Jung et al. [99] for cluster miR-17-92. Finally, Chen et al. [100] have revealed that miR-122 downregulates HBV replication by binding to the viral target sequence contributing to chronic HBV infection.

## 5. Human Papillomavirus

The human papillomaviruses (HPVs), double-stranded DNA tumor viruses, belong to the family *Papillomaviridae*. HPVs infect epithelial cells of the skin or mucosa and may cause benign proliferations such as papillomas or warts. Further, some types of HPVs also have an oncogenic potential and are the main etiologic factor or cofactor in a variety of carcinomas. HPVs are the most frequent viral agents involved in oncogenesis attributable to infectious agents, with almost 640,000 new cases in 2012 [1]. HPVs cause almost 100% of cervical cancers. HPVs participate in oncogenesis in other anogenital regions, such as vulvar, penile, or anal area, and in the development of head and neck cancer; however, the global burden of HPV-associated cancer in these anatomical locations is substantially lower [1].

The genome of HPV is composed of early-region genes *E1–E7*, a late region with two capsid proteins L1 and L2, a long control region (LCR) with regulatory sequences, and a viral origin of replication. Some types of HPV also express the E8^E2C fusion protein [101,102,103]. Viral early-region proteins E6 and E7 are the main oncoproteins in HPVs, and their overexpression contributes to tumor development. The E5 viral protein has a high transformation potential in bovine papillomavirus 1 (BPV1) [104]. In HPVs, the presence of *E5* in the viral genome correlates with the risk of cancer, and E5 cooperates with the main viral oncoproteins E6 and E7 [105,106]. Protein E6 inactivates the function of tumor suppressor p53 [107,108], while protein E7 binds the retinoblastoma protein Rb and thus activates cell cycle progression [109]. The virus might incorporate into the host genome and thus enhance the malignant progression. The integration is an important event in HPV-related carcinogenesis, whose frequency depends on the stage of disease, HPV type, and type of HPV-associated tumor [110], but it is not obligatory for cell transformation.

Profiling of miRNA expression in HPV-associated malignancies has been done in numerous studies with the aim to define new biomarkers for the detection of premalignant stages of the disease as well as markers for selection of patients for modified treatments [111]. Since HPV-associated tumors are etiologically distinct from viral-unrelated tumors of the same anatomical location, for the interpretation of the data, it is important whether the studies also specify the viral status. For cervical cancer, numerous such studies have been published [112,113,114,115], while for head and neck tumors, the information about the viral status has not been commonly evaluated [111,116,117,118,119,120,121]. The knowledge about tumor viral status is specifically very important for head and neck tumors where only 20–90% of them are HPV-associated [122,123].

The process of how the HPV oncoproteins affect the expression of cellular miRNAs was studied by Harden et al. [124], who have assumed that the modulation of the cellular miRNA expression is the main oncogenic activity of these proteins and have suggested several mRNA–miRNA pairs as potential drivers of HPV carcinogenesis. The miR-106b~25 cluster is one of those regulated by transcription factors of the E2F family (transcription factors of higher eukaryotes) and thus by HPV E7 [125]. Furthermore, the expression of the miR-15b~16-2 cluster or the miR-34 family is regulated by HPV oncoproteins [126,127] leading to cell cycle progression and contributing to tumor development. The expression of miR-23b has been shown to be downregulated by the action of the viral E6 protein, resulting in an increase in a direct miRNA target, urinary plasminogen activator (uPA), and thus promoting tumor cell migration [128]. The expression of miR-9 has been found upregulated in both cervical and tonsillar tumors [119,121]. The activation of miR-9 increases cell motility and has been shown to be involved in the pathways regulating metastasis [129,130]. Downregulation of miR-218, identified in cervical cancer as well as in head and neck cancers, has been shown to promote cell migration and invasion [131,132]. While the expression of miR-375 was also found downregulated in cervical tumors [133], it functions as a tumor suppressor by targeting HPV transcripts. This leads to the repression of the expression of E6/E7, cell cycle arrest, and reduced proliferation of HPV-positive cervical cancer cells.

## 6. Merkel Cell Polyomavirus

Polyomaviruses are nonenveloped DNA viruses belonging to the family *Polyomaviridae* which infect a number of hosts, such as birds or mammals. It was long considered that humans could only be infected by JC and BK polyomaviruses; nevertheless, up to now, at least 11 more human polyomaviruses have been identified, including Merkel cell polyomavirus (MCPyV). MCPyV was discovered to be associated with human cancer in 2008 [134], and since then, the interest in polyomavirus research has increased. Whereas MCPyV was detected in more than 90% of Merkel cell carcinomas (MCCs) [135,136], the oncogenic properties of JCPyV and BKPyV have only been documented in cell cultures and animal models [137]. Only scarce studies suggest their oncogenic potential in humans [138,139,140].

The oncoproteins that drive the virus-mediated oncogenesis are early-coded large T (LT) antigen and small T (ST) antigen [141]. Besides these early antigens, MCPyV encodes two more early proteins, the 57kT antigen and alternative LT open reading frame (ALTO), and three late-coded proteins, VP1-3. The first polyomaviral miRNA was described in polyomavirus SV40 [142]; it is encoded on the 3′ end of the late transcript and is complementary to early viral mRNAs. The authors have shown that it reduces the cytotoxic T-lymphocyte-mediated cell lysis and interferon gamma (INF-γ) release. Later, JCV-miR-J1 has been identified in JCPyV and BKV-miR-B1 in BKPyV [143]. These two miRNAs function as inhibitors of NK-cell response [144]. In 2009, MCV-miR-M1 was discovered in MCPyV [145]. All these viral miRNAs influence the expression of LT antigen and thus regulate the viral life cycle and contribute to the host immune evasion.

As documented in previous chapters, miRNAs encoded by oncogenic viruses contribute to the tumorigenesis; however, no human polyomaviral miRNAs have yet been implicated in this process. In tumors, MCPyV miRNAs are not detected or are detected in very low levels (<0.025%) [146,147], because MCPyV DNA is integrated into the host genome in most tumors [134,148]. Therefore, only early genes are expressed, whilst PyV miRNAs are encoded from the late transcripts that are expressed during lytic infection. However, this is in contrast with the study of Chen et al. [149], who chose phylogenetically-related raccoon PyV (RacPyV) to investigate the function of PyV miRNA in tumors. Despite the genomic and sequence similarities of RacPyV miRNA with MCPyV miRNA, high levels of early gene transcripts and miRNA levels have been detected in RacPyV-associated tumors [149,150]. Thus, their observations suggest that these PyV-associated tumors arise via different mechanisms, and MCPyV miRNA probably is not involved in MCC tumorigenesis. Therefore, only future research will reveal the role of PyV miRNAs in virus-mediated carcinogenesis.

The effect of LT antigen on the cellular miRNA expression has not yet been investigated; however, the control of host miRNA expression by the LT antigen is assumed, since this protein is involved, among others, in transcriptional regulation of cellular genes, affects RNA polymerase II-dependent transcription, and thus, might influence the production of pri-miRNAs [137]. The only study to analyze the cellular miRNA expression profiles was that of Xie et al. [151], who compared the miRNA profiles in MCPyV-positive and MCPyV-negative MCC. As expected, they have revealed distinct patterns and have found miR-203, miR-30a, miR-769-5p, miR-34a, and miR-375 to be significantly deregulated. In addition, the authors tested the functional consequences of the overexpression of miR-203 in MCC and have revealed that it functions as a tumor suppressor since it inhibits the cell growth, induces cell cycle arrest, and regulates survivin expression in MCC cells non-associated with MCPyV [151].

## 7. Hepatitis C Virus

Hepatitis C virus (HCV) belongs to the family *Flaviviridae* and is the only representative of the RNA viruses group that is associated with the development of tumors. HCV affects hepatocytes and causes acute infection, which progresses to chronic disease in 75–80% of cases, thus increasing the risk of cirrhosis and/or hepatocellular carcinoma (HCC) [152]. HCV might also contribute to the development of several other malignancies, such as pancreatic or renal cancer and B-cell non-Hodgkin lymphoma [153]. HCV is the fourth leading infectious agent contributing to carcinogenesis, with almost 8% of new cases of cancer being attributable to infection in 2012 [1]. The HCV genome is a positive RNA strand which encodes structural proteins (Core, E1 and E2) and nonstructural proteins (NS2, NS3, NS4A, NS4B, NS5A and NS5B). HCV-associated oncogenesis is promoted by both direct and indirect mechanisms [154]. The direct mechanisms involve primarily the activity of viral proteins, such as core and nonstructural proteins, that promote tumorigenesis through interaction with cell factors leading to the activation of tumorigenic pathways and cell transformation, and the indirect mechanisms then include the promotion of inflammation and oxidative stress.

There is no evidence that HCV encodes its own miRNAs. This is probable because of the separation of the first step of the miRNA biogenesis in the nucleus and replication of the RNA viruses in the cytoplasm where they are physically separated from the nuclear Drosha enzyme and thus cannot undergo the splicing process to generate pre-miRNAs. Still, some groups searched for HCV-encoded miRNAs, but with no success [155]. Nevertheless, HCV infection is associated with changes in cellular miRNA expression, and also, cellular miRNAs influence the HCV life cycle.

An important role in HCV infection and life cycle is played by liver-specific cellular miR-122, whose downregulation results in a decrease in the HCV RNA level [156,157,158]. This miRNA has a binding site in the 5′ UTR of the HCV genome and, interestingly, its binding to the target site does not lead to the repression of HCV genes, but miR-122 protects the HCV genome from nucleolytic degradation and, in this way, promotes viral RNA stability. This miRNA is a very promising candidate for the anti-HCV treatment. Its inhibition by antisense oligonucleotides has been tested in animals [159], and it is now in clinical trials for the treatment of HCV infection [160]. Apart from miR-122, other miRNAs were found to inhibit HCV replication and therefore are potential targets for anti-HCV therapy. MiR-199a targets the 5′ UTR of the HCV genome [161] and appears to be decreased in HCC [162]. MiRNA let-7b binds to two conserved regions in the HCV genome, 5′ UTR and NS5B and besides the inhibition of viral replication, it acts synergistically with IFNα [163]. Mukherjee et al. [164] have shown that miR-181c binds to the E1 and NS5A sites in the HCV genome and reduces viral replication. However, still more research is needed, since the efficiency of miR-181c binding differs between HCV genotypes. The HCV life cycle can also be regulated by host miRNAs that target host mRNA. MiR-373 facilitates HCV RNA replication through the regulation of the JAK/STAT signaling pathway [165], and miR-27a influences the production of HCV particles by inhibiting genes related to the lipid metabolism signaling pathways [166].

There are many studies reporting deregulated expression of cellular miRNAs in HCV-associated tumors. Zhang et al. [167] have observed the upregulation of miR-155 during HCV infection. This miRNA promotes the proliferation of hepatocytes by the activation of Wnt signaling and inhibits apoptosis. Moreover, the growth, proliferation and tumorigenesis of hepatocytes are facilitated by the downregulation of miR-152 [168], miR-181c [164], or miR-491 [169]. The direct role of miR-141 has been shown by Banaudha et al. [170], who found miR-141, required for HCV replication, to inhibit tumor suppressor gene *DCL-1* (Dicer-like 1) which encodes a Rho GTPase-activating protein, and thus promotes the cell proliferation. Varnholt et al. [171] have examined the miRNA expression profiles in a set of liver tumors and dysplastic samples. They have revealed 10 upregulated and 19 downregulated miRNAs in tumors compared to normal tissues. Moreover, they validated five of these miRNAs on a larger set of samples and found the well-known miR-122 to be overexpressed, as was also the case with miR-100 and miR-10a, whereas the expression of miR-198 and miR-145, considered as tumor suppressors, was significantly decreased in tumors. Ura et al. [172] performed a study comparing miRNAs in HBV- and HCV-associated HCC and have revealed that in HCV-related HCC, the differentially deregulated miRNAs are linked to the regulation of the immune response, antigen presentation, cell cycle, or lipid metabolism, while in HBV-associated HCC, they are rather involved in the pathways regulating cell death, DNA damage or signal transduction. Bandiera et al. [173] performed a profiling analysis and have revealed 72 miRNAs to be deregulated more than two-fold in HCC. They further focused on miR-146a-5p, which positively influences the HCV replication and increases HCV infection. Finally, a comprehensive study was done by Pineau et al. [174], who analyzed 104 HCC cases, 90 cirrhotic livers, 21 normal tissues and 35 HCC cell lines, and have identified 12 miRNAs (miR-106b, miR-21, miR-210, miR-221, miR-222, miR-224, miR-34a, miR-425, miR-519a, miR-93, miR-96 and let-7c) as linked to disease progression and tumorigenesis, with four of them (miR-21, miR-221, miR-222 and miR-224) being previously reported as deregulated in HCC.

## 8. Retroviruses

Retroviruses are a group of single-stranded RNA viruses that use their own encoded reverse transcriptase to produce DNA intermediate from their RNA genome, and then the virus integrates into the genome of the host and is transcribed and translated along the cellular genes. Retroviruses include tumor-associated viruses infecting animals, such as Rous sarcoma virus or mouse mammary tumor virus (MMTV). The malignant transformation is caused by viral proto-oncogenes or through the disruption or activation of cellular proto-oncogenes. The most important tumorigenic retrovirus of humans is human T-cell lymphotropic virus 1 (HTLV-1) from the genus *Deltavirus*, which is linked to the type of lymphocytic leukemia and non-Hodgkin lymphoma called adult T-cell leukemia/lymphoma (ATL). HTLV-1 was responsible for only 0.1% of new cancer cases attributable to infectious agents in 2012, with around 3000 new cases reported worldwide [1]. HTLV-1 encodes the oncogenic protein Tax, which interacts with more than one hundred cellular proteins and prevents apoptosis, enhances cell signaling, induces cell cycle dysregulation, or activates cellular proto-oncogenes. The second important oncogenic protein is HTLV-1 bZIP factor (HBZ), which is present in 100% of ATL cells, enhances T-cell proliferation, and contributes to the prevention of apoptosis [175]. The human immunodeficiency virus (HIV), a member of the genus *Lentivirus*, causes acquired immune deficiency syndrome (AIDS). This virus does not appear to induce cancer directly, but increases the risk of the development of other viruses-related tumors, such as Kaposi’s sarcoma, cervical cancer, or non-Hodgkin lymphoma.

There are several studies focused on cellular miRNA targets in the retroviral genome and the influence of their binding on viral replication, gene expression, or infectivity. Huang et al. [176] have shown that the 3′ end of the HIV mRNA in resting CD4+ T-cells is targeted by cellular miR-28, miR-125b, miR-150, miR-223 or miR-382, and contributes to HIV-1 latency. Two computational studies were conducted to find potential target sites of cellular miRNAs in HTLV-1 [177,178]. Bai and Nicot [179] have found that miR-28-3p inhibits HTLV-1 replication and expression by targeting a specific site within gag/pol in viral mRNA, and have identified a mechanism of how cellular miRNA prevents viral transmission. Besides the direct targeting of the viral genome, viral replication can also be influenced by cellular miRNAs indirectly through host-dependency factors (HDFs) [180]. For example, miR-20a and miR-17-5p target the p300/CBP-associated factor important for long terminal repeat (LTR) activation, and the upregulation of these miRNAs reduces HIV-1 replication [181]. Further, the overexpression of miR-198 and miR-27b leads to the repression of cyclin-T1, a cofactor of Tat, and thus to HIV inhibition [182,183]. On the other hand, miR-217 and miR-34a target sirtuin 1 (SIRT1), which disrupts LTR activation by Tat, leading to HIV transactivation [184].

Similarly to other oncoviruses, retroviruses also dysregulate the expression of cellular miRNAs. Van Duyne et al. [185] have shown that HTLV-1 infection significantly downregulates Drosha protein expression, which is required for the cleavage of pri-miRNA, by direct interaction with the Tax protein, and thus deregulates the miRNA biogenesis pathway. Several studies have characterized the miRNA expression profiles in HTLV-1-transformed cell lines or ATL patients. Pichler et al. [186] have demonstrated deregulation of five miRNAs (miR-21, miR-24, miR-146a, miR-155 and miR-223) in HTLV-1-transformed T-cells. Moreover, they have found that miR-146a is transactivated directly by the Tax protein via NF-κB signaling, and its upregulation promotes HTLV-1 T-cell proliferation. The analysis of miRNA expression of HTLV-1 transformed T-cell lines and ATL patients has revealed six miRNAs to be consistently upregulated, with two of them (miR-93 and miR-130b) targeting the 3′ UTR of tumor suppressor protein TP53INP1 (TP53-induced nuclear protein 1), impacting proliferation and survival of HTLV-1-transformed/infected cells [187]. Bellon et al. [188] analyzed the miRNA profiles of ATL patients with microarrays, reporting deregulation of several miRNAs. However, they have also observed that two miRNAs (miR-150 and miR-223) are differentially expressed, both in vitro and ex vivo. Similarly, Yamagishi et al. [189] performed microarray analysis of ATL cells from patients and, interestingly, have revealed downregulation of 59 miRNAs out of 61, with miR-31 being the most repressed. MiR-31 is reported as a tumor suppressor regulating the NF-κB signaling pathway, and its deregulation leads to resistance of cells to apoptosis. The cellular miRNA expression can also be influenced by another retroviral oncoprotein, HBZ, which activates miR-17 and miR-21 in CD4+ T-cells [190]. These miRNAs target DNA-damage factor OBFC2A and thus promote cell proliferation and genomic instability.

## 9. Controversial Oncoviral-Encoded miRNAs

Besides approved and validated oncoviral-encoded miRNAs, the existence of viral-specific miRNAs from other groups of human oncoviruses has been reported in the literature. However, for these miRNAs, the evidence of their miRNA biogenesis as well as their function and clinical significance is not in the literature adequately supported. Therefore, further research is needed.

The existence of HBV-encoded miRNAs was not experimentally confirmed until recently. There was only one study, that of Jin et al. [191], who analyzed candidates for viral-encoded miRNAs in silico. They found only one pre-miRNA candidate for which one target viral mRNA was revealed but was not confirmed in vivo. No cellular mRNA was found as a target of this predicted viral miRNA. Recently, Yang et al. [192] performed a study of HBV-encoded miRNAs by deep sequencing and Northern blotting. Although they did not find the previously computationally predicted miRNA [191], they identified five small noncoding RNAs (snRNAs) aligning to HBV transcripts, and validated a novel HBV-miR-3, which they suggested, is involved in the viral replication process and represses HBV protein expression and virion production, probably contributing to the establishment of persistent infection. However, the number of reads obtained by deep sequencing in their study is not compelling, since it makes up only <0.0004% of the standard number of NGS reads. Moreover, the predicted secondary structure of pre-miRNA also does not fulfil the criteria for authentic viral miRNAs and natural substrate for enzymes of miRNAs biogenesis. Furthermore, they showed that the biogenesis of this HBV-encoded snRNA proceeds via the classical Dicer and Drosha route [192]. This is contradictory to results published by Wang et al. [193], who demonstrated that HBV-derived snRNAs are not processed by Dicer. Additionally, several studies analyzed the expression of snRNAs in hepatocellular carcinomas associated with HBV by deep sequencing [162,194,195,196]. Even though these studies were focused primarily on cellular miRNAs, we assume that any HBV viral miRNAs markedly differentially expressed would have been noticed in these studies. Thus, these controversial data highlight the fact that further research on HBV-encoded miRNAs is needed.

The second group of oncoviruses with not-definitely-approved viral-encoded miRNAs are HPVs. It is generally accepted that HPVs do not encode their own miRNAs; nevertheless, results of two research groups have suggested the existence of HPV-encoded miRNAs. First, the group of Auvinen has reported the identification and validation of the first papillomavirus-encoded miRNAs in human cervical lesions and in cell lines using SOLiD sequencing, quantitative RT–PCR, and in-situ hybridization [197]. They have successfully validated four miRNAs, and based on the prediction of target genes, they have suggested their role in cell cycle regulation, immune functions, cell adhesion and migration, development, and cancer. Several years later, the same group performed the analysis of the reported miRNAs in cervical samples by quantitative RT–PCR, and they detected low levels of expression of these miRNAs in all cases [198]. Finally, Weng et al. [199] have developed a systematic method for viral miRNA identification and regulatory network construction based on genome-wide sequence analysis, and have predicted other putative miRNAs by bioinformatics approaches. The target genes of these predicted miRNAs play roles in virus infection and carcinogenesis and might be possible targets for antiviral drugs. However, no studies of in-vivo function of the predicted HPV-specific miRNAs have been published so far.

The topic of miRNAs encoded by retroviruses is also still quite controversial. One of the reasons is the risk of cleavage of the viral RNA genome with Rnase Drosha during the biogenesis of miRNAs. However, two groups have reported studies of in-vitro Dicer processing of ~50-nt long HIV-1 TAR RNA, which forms a stem–loop structure similar to pre-miRNA [200,201]. Moreover, Rouha et al. [202] have revealed that miRNAs might be produced by RNA viruses replicating in the cytoplasm without impairing viral RNA replication. Recently, Harwig et al. [203] suggested that the HIV-1 TAR hairpin structure could be a source of miRNAs without cleavage of the RNA genome, however, the biogenesis differ from the canonical miRNA pathway. Currently, the miRNA database (available online: http://mirbase.org) indicates that HIV-1 encodes three precursors of miRNAs and four mature miRNAs, however, they should be referred to as small noncoding RNAs (snRNAs) since there is a lack of significant experimental support demonstrating that these are functional miRNAs that arise from a stable hairpin RNA structure and are processed by the classical miRNA biogenesis pathway. Bernard et al. [204] have reported that HIV-1 produces the viral miRNAs vmiR-88, vmiR-99 and vmiR-TAR, with the first two stimulating human macrophage TNFα release and contributing to chronic immune activation; however, in-vivo confirmation of their in-vitro results remains to be determined. Moreover, Li et al. [205] have detected vmiR-TAR-3p in primary macrophages infected by HIV, and have shown its inhibitory effect on the viral replication. Klase et al. [206] and Ouellet et al. [207] have reported the anti-apoptotic role of vmiR-TAR-5p and vmiR-TAR-3p. Further, hiv1-miR-N367, described within the *nef* gene, targets the Nef protein for degradation, which may play a role in the establishment of viral latency [208,209]. Finally, hiv1-miR-H1, identified in 3′ LTR, targets the gene for apoptosis antagonizing transcription factor (AATF), leading to an increase in cell apoptosis [210], and hiv1-miR-H3 has been reported to target 5′ LTR within the TATA box increasing the promoter activity and positively regulating the viral replication [211]. Unlike for HIV, miRNAs encoded by HTLV-1 have not yet been identified. Despite these findings, various reports have been published where the authors failed to detect HIV-1 miRNAs, and/or the candidate miRNAs failed to satisfy all defined criteria for authentic viral miRNAs [155,212,213]. Lin et al. [212] demonstrated that neither HIV-1 nor HTLV-1 express significant levels of small interfering RNAs (siRNAs) or miRNAs, as well as that they do not repress cellular RNA interference machinery in the infected cells. This was supported by Whisnant et al. [213], who performed deep sequencing analysis of HIV-1 infected cell cultures and revealed only very few reads aligning to the viral genome. In contrast to reads aligning to the human genome that peak at ~22 nt, viral reads showed more reads at smaller size. Moreover, matching of HIV-1-derived snRNAs to the proviral genome was scattered over the proviral sequence, and their 5′ ends did not share the parameters characteristic for the authentic miRNAs. The authors also refuted the TAR-derived miRNAs that are mentioned above [206,207], since the TAR stem–loop differs from the authentic pre-miRNAs. Finally, they demonstrated [213] that HIV-1-derived snRNAs were not loaded into RNA-induced silencing complex (RISC), and further, they failed to detect a significant level of small RNA reads derived from the HIV-1 antisense strand, which was previously reported by Schopman et al. [214]. Similarly, Vongrad et al. [215], using high-throughput methods, failed to detect the incorporation of HIV-1-derived sncRNA or HIV-1 target sequences into the AGO2-RISC complex of RNAi pathway. However, it is worth mentioning the observation made by Kincaid et al. [216], who found out that bovine leukemia virus (BLV) from the family *Retroviridae* encodes a conserved cluster of miRNAs. They further show that one of these viral miRNAs shares a partial sequence with cellular miR-29, whose overexpression is associated with B-cell neoplasm. This type of malignancy resembles tumors initiated by BLV, and therefore, their findings suggest the possible way how this BLV-associated miRNA can contribute to tumorigenesis. Therefore, despite the numerous studies that support the nonexistence of HIV-1-specific miRNAs, the findings of bovine leukemia virus-specific miRNAs pointed to the importance of further research in this area.

## 10. Concluding Remarks

**Figure 1:**
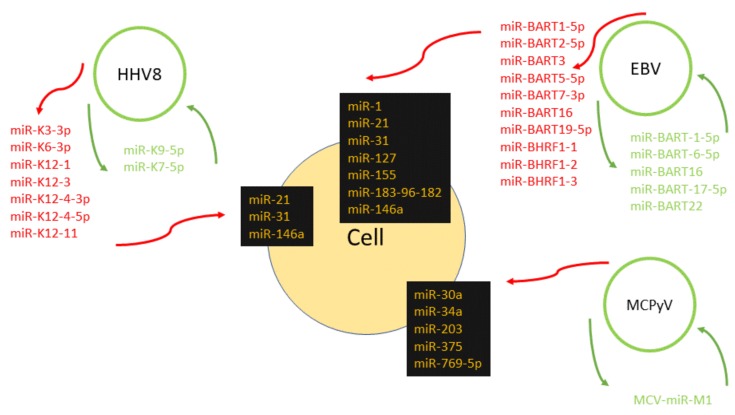
Viral miRNAs of Epstein–Barr virus (EBV), human herpesvirus 8 (HHV8) and Merkel cell polyomavirus (MCPyV), and cell miRNAs whose expression is influenced by the viral infection. The deregulation of these miRNAs contributes to the transformation of the cell and to tumor development. Green = viral miRNAs that target viral mRNAs, red = viral miRNAs that target cellular mRNAs, and yellow = cellular miRNAs influenced by the viral infection.

Carcinogenic infections are the cause of 15% of tumors worldwide. Tumors related to oncoviruses are studied extensively with the aim to detect new diagnostic and prognostic markers and treatment targets. This review provides a comprehensive overview on the mechanisms of the contribution of human oncoviruses to tumor development, and describes how oncoviruses utilize their own encoded sequences, miRNAs, for the regulation of their gene expression, as well as to influence the gene expression of their hosts (summarized in Table 1 and Figure 1). Besides the expression and function of miRNAs encoded by some groups of oncoviruses, the existence of virally coded miRNAs in several groups of human oncoviruses remains controversial. However, the miRNA regulation is reciprocal and the viral life cycle can also be influenced by cellular miRNAs. In this review, we summarize the accumulated body of knowledge in this area and focus on the oncogenesis mediated by EBV, HHV-8, HBV, HPV, MCPyV, HCV, HIV and HTLV-1. The research into miRNA profiling to set a panel of diagnostic miRNAs for specific tumors is of high relevance, and there have been already developed cancer focus panels with differentially expressed miRNAs for breast cancer, prostate cancer or lung cancer. MiRNAs could be a promising tool for early diagnosis of virus-related tumors and their noninvasive treatment. Currently, some miRNA-based therapies are being tested in preclinical and clinical trials, for example, miR-122 in HCV infection, miR-34 in liver cancer, or miR-208 in cardiometabolic disease [217]. Despite these achievements, additional research is needed for a more precise understanding of the miRNA pathways, including those that are virus associated.

## Figures and Tables

**Table 1:** Summary of authentic viral-encoded miRNAs mentioned in the review, and their viral and cellular targets.

Virus Family	Virus Species	Mature miRNAs (According to miRBase, Updated 2014)	MiRNAs Mentioned in This Review	Proposed Function	Target	Reference
*Herpesviridae*	Epstein–Barr virus (EBV)	44	miR-BART17-5p	cell transformation	LMP1	[25]
			miR-BART16	cell transformation, anti-apoptotic role	LMP1, Casp3	[25,31]
			miR-BART1-5p		LMP1, Casp3	[25,31]
			miR-BART5-5p		PUMA	[29]
			miR-BART19-5p		PUMA	[29]
			miR-BART22	escape from host immune surveillance	LMP2A	[26]
			miR-BART2-5p	regulation of latent–lytic switch, evasion of the host‘s immune system	BALF5, MICB	[27,36]
			miR-BART6-5p	regulation of viral replication	EBNA2	[28]
			miR-BART7-3p	promotion of EMT and metastasis, regulation of radiation sensitivity	PTEN, GFPT1	[30,38]
			miR-BART3	proliferation and cell transformation	DICE1	[32]
			miR-BHRF1-1	immunomodulatory function	CXCL11	[33]
			miR-BHRF1-2		CXCL11	
			miR-BHRF1-3		CXCL11	
	Herpesvirus-8 (HHV-8)/Kaposi’s sarcoma herpesvirus (KSHV)	25	miR-K9-5p	regulation of lytic induction	RTA	[54,55]
			miR-K7-5p			
			miR-K3	regulation of viral latency and angiogenesis	nuclear factor I/B, GRK2, THBS1	[56,62,63]
			miR-K12-11		MYB, IKKε, THBS1	[58,60,62]
			miR-K12-4	regulation of viral latency, anti-apoptotic role	Rbl2, Casp3	[61,67]
			miR-K6-3p	regulation of angiogenesis	THBS1, SH3BGR	[62,63]
			miR-K12-1	anti-apoptotic role, regulation of angiogenesis	p21, Casp3, THBS1	[62,66,67]
*Polyomaviridae*	Merkel cell polyomavirus (MCPyV)	1	MCV-miR-M1	regulation of viral lifecycle	early viral transcripts	[145]

## Glossary

Immortalization of the host cell	continual proliferation of the cell mostly caused by mutation or by the activity of the viral oncogenes
Transformation of the host cell	morphologic, physiologic and genetic changes of the cell initiating a process of tumor development and progression
